# First person – Shrena Chakraborty

**DOI:** 10.1242/bio.061853

**Published:** 2024-12-30

## Abstract

First Person is a series of interviews with the first authors of a selection of papers published in Biology Open, helping researchers promote themselves alongside their papers. Shrena Chakraborty is first author on ‘ The fission yeast SUMO-targeted ubiquitin ligase Slx8 functionally associates with clustered centromeres and the silent mating-type region at the nuclear periphery’, published in BIO. Shrena conducted the research described in this article while a PhD student in the lab of Dr Sarah Lambert at Institut Curie, Orsay, France, and will soon join the same team as a research engineer, investigating molecular mechanisms in cells using cell biology approaches.


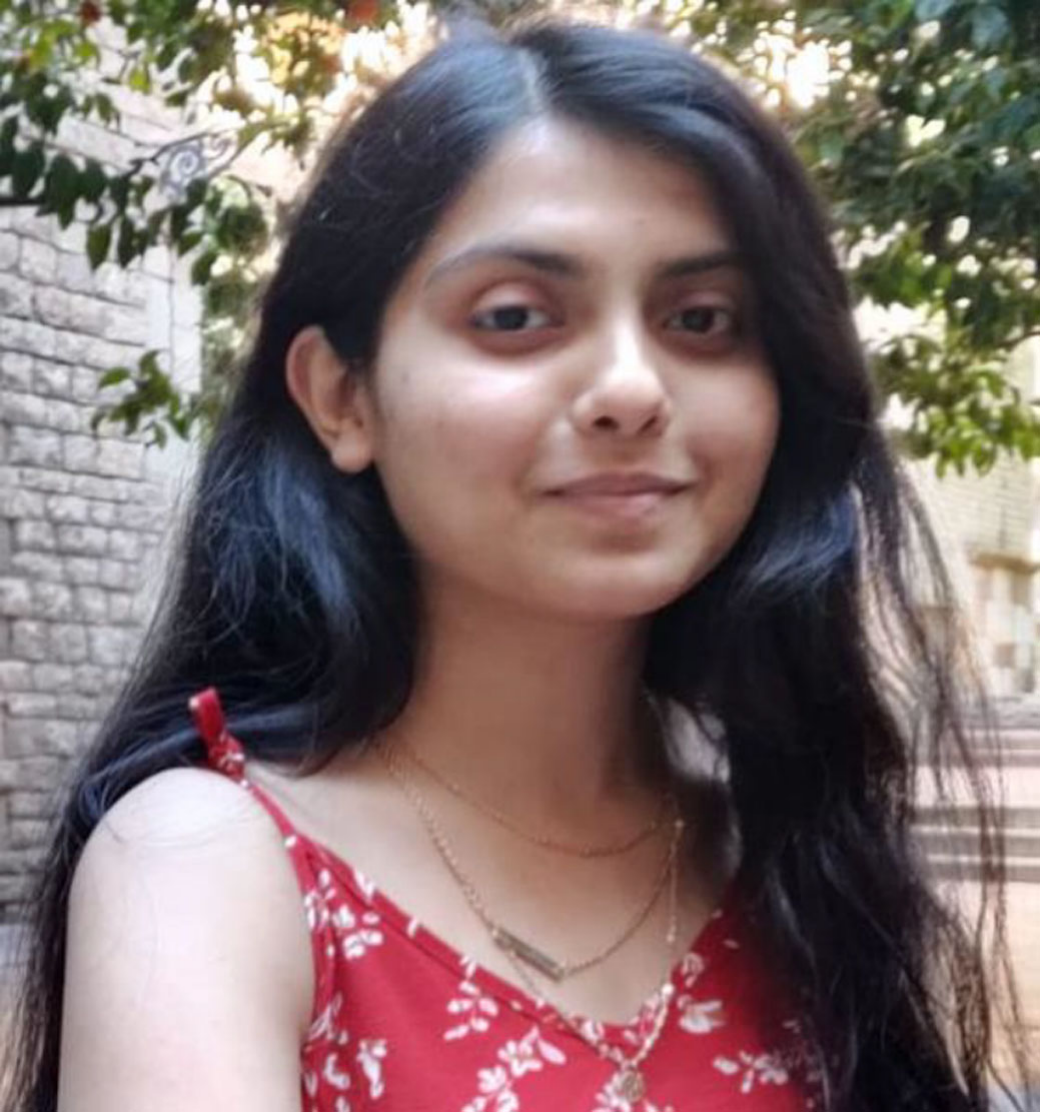


**Shrena Chakraborty**

**Describe your scientific journey and your current research focus**

After defending my PhD degree from Institut Curie, I would like to pursue academia not as a postdoc, but as a research engineer. This position can offer a unique blend of applied research and practical problem solving, often focusing on the development and implementation of innovative technologies and methodologies. Unlike postdoctoral roles, which typically emphasize furthering theoretical research and publishing findings, a research engineer position might involve collaborating on interdisciplinary projects, leading technical teams, and contributing to the advancement of cutting-edge solutions in their field. It is crucial to recognize that this choice reflects a commitment to applying my expertise in concrete ways and contributing to real-world applications of research. It is encouraging for me to explore opportunities that align with my interests, such as research engineering roles in academic labs or collaborative research institutes, which will help me leverage my PhD training effectively while pursuing my career goals.

**Who or what inspired you to become a scientist?**

My sister, who was pursuing her PhD at the time, initially inspired me to become a scientist. Her enthusiasm for sharing the daily challenges and triumphs of academic life deeply resonated with me. As I embarked on my own PhD journey, I came to understand that the path is not always straightforward or easy, but the rewards at the end make it worthwhile. This realization continues to inspire me to persevere through challenges.

This research shows how Slx8 helps organize the cell's DNA and control which parts are active or silent.

**How would you explain the main findings of your paper?**

The STUbL family of proteins helps keep our DNA safe and organized by managing how different parts of the cell work together. One important job they do is moving damaged DNA to specific areas of the cell where it can be repaired. In a type of yeast called *Schizosaccharomyces pombe*, a protein called Slx8 helps the cell handle stress when copying DNA by controlling other proteins.

We studied where Slx8 is located in the cell to understand its role better. To our surprise, we didn't see Slx8 forming clusters when the cell was stressed during DNA copying. Instead, we found it forming one large cluster near the edge of the nucleus. This cluster is connected to key parts of the DNA: the centromeres (which are important for separating chromosomes during cell division) and areas of DNA that stay inactive.

We also found that forming this Slx8 cluster depends on other proteins and specific chemical changes to DNA and proteins. Slx8 helps keep centromeres grouped together and ensures that certain parts of the DNA stay inactive. This research shows how Slx8 helps organize the cell's DNA and control which parts are active or silent.

**What are the potential implications of this finding for your field of research?**

The potential impact of my work lies in uncovering how SUMOylation influences the structure and function of the centromere. A key focus is determining whether SUMO chain formation shows a preference for the centromere or the mating-type locus, and how this contributes to maintaining the stability and organization of these crucial genomic regions.

[…] research doesn't always follow the path you plan, and that's the true beauty of science.

**Figure:**
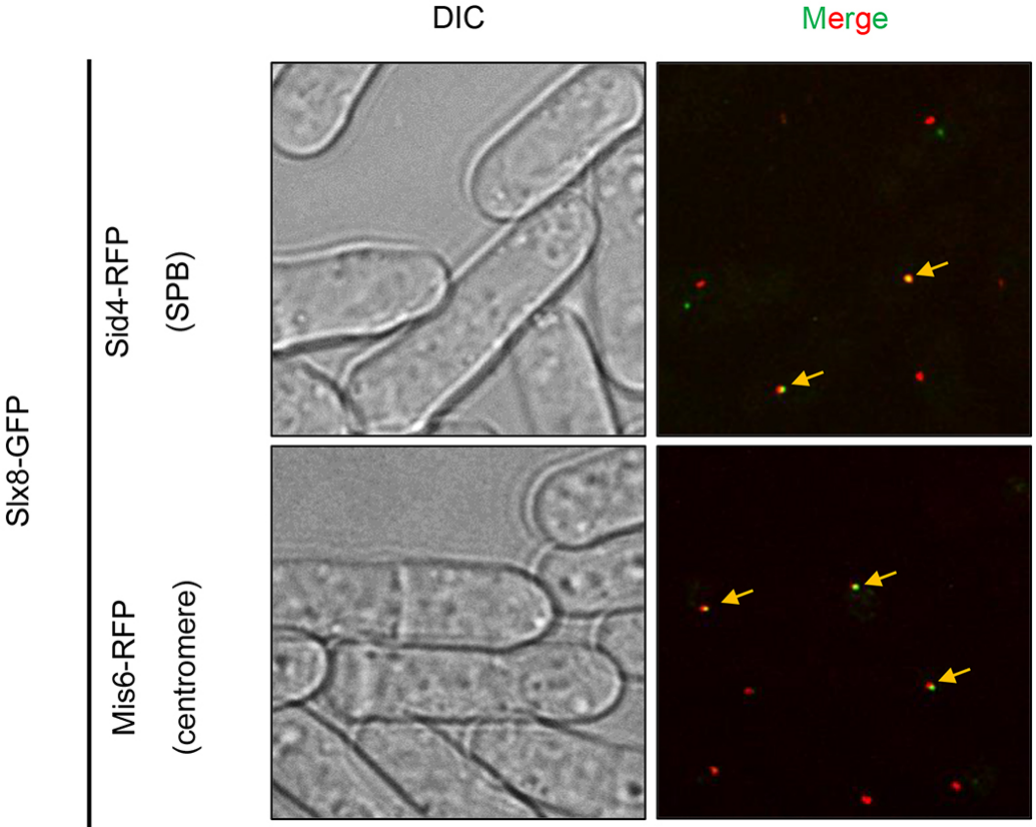
Slx8, SUMO-targeted ubiquitin ligase marks the centromere in fission yeast.

**Which part of this research project was the most rewarding?**

The most rewarding part of the project was the opportunity to restructure it and uncover a completely different angle of discoveries than what I initially expected. This experience taught me that research doesn't always follow the path you plan, and that's the true beauty of science.

**What do you enjoy most about being an early-career researcher?**

What I enjoy most about being an early-career researcher is the flexibility to explore new fields. If something else catches my interest, I still have the opportunity to shift gears, dive into a different area, and embrace new ideas and techniques.

**What piece of advice would you give to the next generation of researchers?**

The most important advice is to stay patient and focused on your goal. Research can be challenging, and some days will be harder than others. However, by staying committed to your purpose, you can achieve outcomes that will both surprise and excite you.

**What's next for you?**

My immediate plan is to join the same team where I completed my PhD as a research engineer. In the long term, I aspire to lead a project with a small team or contribute to such a team in any capacity.
